# Clinical Effectiveness and Safety of Multimodal Prehabilitation in Colorectal Cancer Surgery—A Narrative State-of-the-Art Review of Research and Clinical Trials

**DOI:** 10.3390/jcm15114128

**Published:** 2026-05-27

**Authors:** Dorota Zierkiewicz, Stanisław Manulik, Anna Chudiak, Dorota Krówczyńska, Wojciech Homola, Piotr Pobrotyn, Sylwia Benirowska-Pomianowska, Mariola Głowacka, Małgorzata Paprocka-Borowicz, Mariusz Chabowski

**Affiliations:** 1 Division of Anesthetic and Surgical Nursing, Faculty of Nursing and Midwifery, Wroclaw Medical University, 51-618 Wroclaw, Poland; dorota.zierkiewicz@umw.edu.pl (D.Z.); anna.chudiak@umw.edu.pl (A.C.); 2 Department of Gastrointestinal Cancer, Lower Silesian Oncology, Hematology and Pulmonology Center, 53-413 Wroclaw, Poland; 3 Division of Healthcare Organization, Department of Nursing, Faculty of Nursing and Midwifery, Wroclaw Medical University, 51-618 Wroclaw, Poland; stanislaw.manulik@umw.edu.pl; 4 Cardinal Stefan Wyszynski Institute of Cardiology in Warsaw, 04-628 Warsaw, Poland; dkrowczynska@o2.pl; 5 Department of Nursing and Obstetrics, Collegium Mazovia, 08-110 Siedlce, Poland; 6 Department of Nursing, Faculty of Rehabilitation, Józef Piłsudski University of Physical Education in Warsaw, 00-968 Warsaw, Poland; 7 Gynaecology and Obstetrics Centre FemiMea, 55-040 Bielany Wroclawskie, Poland; wojtek.homola@gmail.com; 8 Department of Clinical Neurosciences, Faculty of Medicine, Wrocław University of Science and Technology, 51-377 Wroclaw, Poland; 9 Department of Nursing, Faculty of Health Sciences, The Mazovian University in Płock, 09-402 Płock, Poland; s.benirowska@mazowiecka.edu.pl (S.B.-P.); m.glowacka@mazowiecka.edu.pl (M.G.); 10 Department of Neurological Rehabilitation, Regional Specialist Hospital in Wroclaw, 51-128 Wroclaw, Poland; malgorzata.paprocka-borowicz@wssk.wroc.pl; 11 Department of Surgery, 4th Military Teaching Hospital in Wroclaw, 50-981 Wrocław, Poland; mariusz.chabowski@pwr.edu.pl; 12 Department of Clinical Surgical Sciences, Faculty of Medicine, Wroclaw University of Science and Technology, 51-377 Wroclaw, Poland

**Keywords:** colorectal cancer, prehabilitation, multimodal prehabilitation, ERAS, perioperative rehabilitation, functional capacity, postoperative complications

## Abstract

**Background/Objectives**: Colorectal cancer (CRC) remains one of the most common malignancies worldwide. Despite advances in surgical techniques and the implementation of Enhanced Recovery After Surgery (ERAS) protocols, a substantial proportion of patients continue to experience postoperative complications and persistent functional impairment. Multimodal prehabilitation, combining physical exercise, nutritional optimization, and behavioral support, has been proposed as a strategy to enhance functional reserve prior to surgical treatment. The aim of this review was to provide a critical narrative evaluation of current clinical evidence regarding the effectiveness and safety of prehabilitation in patients with CRC. **Methods**: A targeted literature review was conducted in PubMed, Embase, CINAHL, Scopus, and Web of Science (2015–2025), identifying 23 primary studies, including 18 randomized and 5 non-randomized trials. **Results**: Most multimodal prehabilitation programs resulted in significant improvements in functional capacity prior to CRC resection. Functional outcomes were most commonly assessed using the six-minute walk test, cardiopulmonary fitness parameters, and measures of muscle strength, with good tolerance and high patient adherence. Several studies also demonstrated reductions in postoperative complication rates, shorter hospital stays, and faster recovery of independence, particularly among older patients, those with frailty, or reduced baseline functional capacity, although these effects were not fully consistent across studies. **Conclusions**: Available clinical evidence supports multimodal prehabilitation as a safe and feasible strategy that consistently preserves and enhances functional reserve prior to CRC surgery. However, its capacity to modify hard perioperative outcomes (such as complication rates and length of stay) remains inconsistent across current RCTs, showing clear efficacy predominantly in targeted high-risk, frail, or malnourished cohorts.

## 1. Introduction

Colorectal cancer (CRC) remains one of the most common malignancies worldwide [1] and represents a major public health challenge in Poland [2]. Despite advances in surgical techniques and comprehensive perioperative care protocols, postoperative complications, prolonged hospitalization, and persistent functional impairment continue to substantially affect treatment outcomes, particularly among older patients and those with comorbidities [3].

Prehabilitation is broadly defined as a proactive process that enhances a patient’s functional capacity prior to oncological treatment to buffer against post-surgical decline. When limited to a single modality (e.g., physical exercise), it is termed unimodal; conversely, multimodal prehabilitation simultaneously combines physical exercise and nutritional optimization, while trimodal interventions append structured psychological or behavioral support. This paradigm complements the principles of Enhanced Recovery After Surgery (ERAS), shifting care from a reactive post-operative approach to proactive preoperative stabilization [4,5].

To date, strategies aimed at improving patient recovery have primarily focused on the postoperative period, particularly through rehabilitation programs. However, this phase does not always represent the optimal time for implementing lifestyle modifications, as cancer patients often experience pronounced fatigue, concerns related to wound healing, or anxiety associated with awaiting further treatment of the underlying disease [6].

In contrast, the preoperative period may offer more favorable conditions for intervention, as patients typically demonstrate better physical capacity than in the immediate postoperative phase and often have a relatively extended waiting time before planned surgery. This reflects the fundamental rationale of prehabilitation as a targeted process to improve patients’ functional reserves prior to surgical intervention in order to enhance physiological tolerance to the forthcoming stress associated with oncological treatment [7].

A recently published integrative review by Polish authors addressing prehabilitation prior to chemotherapy further highlights the interval between cancer diagnosis and initiation of systemic treatment as a “therapeutic window” for interventions aimed at improving physical fitness, nutritional status, psychological well-being, and modifying adverse health behaviors. The authors emphasize that despite promising evidence regarding high-protein nutrition, physical activity, psychological support, and integrated behavioral programs, standardized prehabilitation protocols prior to systemic therapy and formal guidelines from scientific societies remain lacking. Moreover, existing literature has predominantly focused on patients with breast cancer, while other malignancies, including CRC, remain relatively underinvestigated. This constitutes a clear research gap and underscores the need for further studies on prehabilitation in the perioperative oncological settings [8].

In recent years, a growing number of clinical studies have been published evaluating the effectiveness and safety of prehabilitation in patients with CRC, including both classical randomized controlled trials (RCTs) as well as pragmatic studies and real-world analyses. These investigations differ substantially in study design, duration of intervention, components of prehabilitation programs, and selection of outcome measures, which complicates direct comparison of results and their straightforward translation into clinical practice. At the same time, emerging evidence suggests that the benefits of prehabilitation may be particularly pronounced in specific patient subgroups, such as older adults, individuals with reduced baseline functional capacity, or those facing a limited preoperative time window.

Within the Polish healthcare system, prehabilitation has recently been clearly defined and structured through interdisciplinary expert recommendations emphasizing the need for comprehensive patient preparation prior to surgical and oncological treatment [9]. These guidelines highlight that despite advances in surgery and anesthesiology, a substantial proportion of patients, particularly older individuals, those with frailty syndrome, malnutrition, or multimorbidity, fail to achieve satisfactory functional recovery without targeted preoperative optimization. Consequently, prehabilitation is increasingly recognized as a key component of modern perioperative care.

Notably, there remains a lack of state-of-the-art reviews that critically and systematically synthesize current clinical evidence on prehabilitation in CRC. In particular, no comprehensive analyses have yet identified consistent patterns of effectiveness, safety, and feasibility across available studies, nor clearly delineated priorities for future research. Therefore, the aim of this narrative review is to present the current state of knowledge based on primary clinical studies investigating the effectiveness and safety of prehabilitation in patients with CRC, with a particular focus on multimodal interventions.

The aim of this article, based on a state-of-the-art narrative review of clinical studies, is to provide a comprehensive and critical synthesis of current evidence regarding the effectiveness and safety of prehabilitation in patients with CRC, with particular emphasis on multimodal interventions. The review focuses on the analysis of clinical, functional, and perioperative outcomes, as well as on the identification of key characteristics of prehabilitation programs that may determine their effectiveness in routine clinical practice.

## 2. Materials and Methods

### 2.1. Review Framework and Search Criteria

This article was prepared as a state-of-the-art narrative literature review, structured and evaluated in accordance with the SANRA (Scale for the Assessment of Narrative Review Articles) guidelines to ensure qualitative methodological rigor [10]. To maintain transparency in the literature selection process, a structured, targeted literature search was conducted across the PubMed/MEDLINE, Embase, CINAHL, Scopus, and Web of Science databases.

The search covered peer-reviewed publications published between January 2015 and December 2025. This design deliberately eschews a formal systematic review or meta-analytical framework in order to leverage a descriptive, interpretive approach capable of synthesizing highly heterogeneous clinical protocols and real-world implementation models.

### 2.2. Search Strategy and Eligibility Criteria

The literature search was executed using explicit Boolean strings tailored to each database. The primary search strings utilized in PubMed/MEDLINE were: * (“prehabilitation” [Title/Abstract] AND (“colorectal neoplasms” [MeSH Terms] OR “colorectal cancer” [Title/Abstract])) (n = 423) * (“prehabilitation” [Title/Abstract] AND (“colorectal cancer” [Title/Abstract] OR “colorectal surgery” [Title/Abstract]) AND (“surgical procedures, operative” [MeSH Terms] OR “surgery” [Title/Abstract])) (n = 414).

Studies were eligible for final narrative synthesis if they met the following predefined criteria: (1) adult patients diagnosed with CRC undergoing elective surgical resection; (2) implementation of a structured prehabilitation intervention (unimodal or multimodal) initiated in the preoperative phase; and (3) reporting of functional, clinical, or perioperative outcomes. Studies were excluded if they were case reports, animal models, letters, or involved non-oncological populations.

### 2.3. Search Results and Qualitative Synthesis

**Figure 1:**
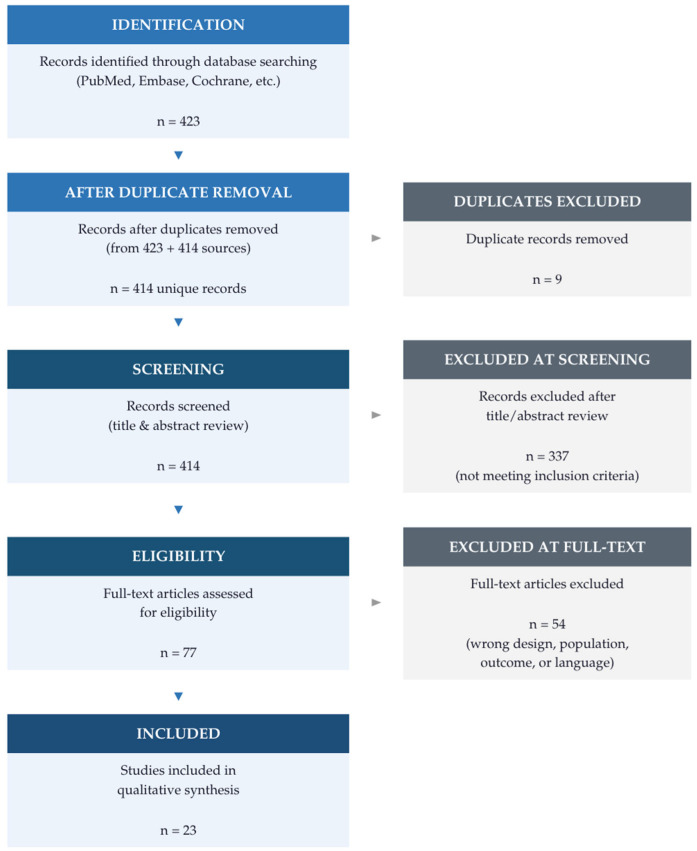
Study selection flow diagram (PRISMA-style).

A total of 23 primary studies were ultimately included in the review, comprising 18 randomized controlled trials (RCTs) and 5 non-RCTs (prospective cohort, observational, and retrospective designs). The study selection workflow detailing initial record identification, duplicate removal, title/abstract screening, and full-text eligibility assessment is visually outlined in the study selection flow diagram (Figure 1).

Consistent with a state-of-the-art narrative review framework, these studies were not subjected to quantitative pooling or formal systematic risk-of-bias grading; instead, they underwent a qualitative narrative synthesis focused on identifying consistent clinical patterns, intervention safety, and practical implications for routine implementation.

## 3. Results

### 3.1. Evidence Overview and Qualitative Critical Appraisal

Summary of evidence on the use of prehabilitation in surgically treated CRC patients based on current clinical and scientific studies is presented in Table 1. Moreover, a detailed summary of all included studies, including intervention characteristics, outcomes, and safety profiles, is provided in Supplementary Table S1.

To contextualize the strength of the synthesized evidence and avoid overinterpreting isolated positive findings, a qualitative critical appraisal of the literature was conducted. The compiled evidence base exhibits substantial methodological heterogeneity. Of the 23 studies, the majority (n = 18) are RCTs, which provide high-level evidence; however, several of these trials [11,12,13] were explicitly designed as small-scale pilot or feasibility studies underpowered to detect significant differences in hard clinical endpoints. Furthermore, 5 studies utilize observational or historically controlled cohort designs, which carry an inherent risk of selection bias [14,15,16,17,18]. Blinding of participants and personnel was absent in nearly all physical training protocols due to the nature of the intervention, with only one single-blind and one double-blind design reported, representing a minor systemic vulnerability across the current literature. Consequently, the descriptive synthesis presented below must be interpreted through the lens of this baseline methodological variance.

Bousquet-Dion et al. [19] performed a prospective RCT in 63 patients undergoing elective CRC surgery, comparing multimodal prehabilitation (n = 37) with postoperative rehabilitation (n = 26). The 3–4-week preoperative intervention combined supervised aerobic and resistance exercise, home-based physical activity, nutritional counseling with whey protein supplementation, and anxiety-reduction strategies within an ERAS pathway. Although no significant between-group difference was found in the primary outcome (6MWT), the prehabilitation group demonstrated higher levels of moderate-to-vigorous physical activity and better preservation of perioperative functional capacity. The program was safe and well tolerated, although the small sample limited statistical power.

Karlsson et al. [11] conducted a feasibility RCT of home-based exercise prehabilitation in 23 older patients (≥70 years) awaiting CRC surgery. The short preoperative program included respiratory training, functional strength exercises, and aerobic activity, with very high adherence (approximately 97% of supervised sessions). Inspiratory muscle strength improved significantly, while no definitive conclusions could be drawn regarding broader clinical outcomes because of the small sample size. The intervention was safe and feasible.

Carli et al. [20] evaluated multimodal prehabilitation in a single-blind RCT involving 110 frail older patients undergoing CRC resection. The approximately 4-week intervention included supervised and home-based exercise, nutritional counseling with protein supplementation, and psychological support within an ERAS pathway. No significant differences were observed between the prehabilitation and postoperative rehabilitation groups in the primary outcome (30-day CCI) or in secondary outcomes such as complications, length of stay, readmissions, functional recovery, or PROMs. However, preoperative walking capacity improved more in the prehabilitation group. The intervention was safe and well tolerated.

Northgraves et al. [12] conducted a feasibility RCT in 22 patients scheduled for elective CRC surgery, testing an individualized supervised exercise program delivered over approximately 3 weeks. The intervention improved TUG, stair climb performance, and 6MWT results, although the study was underpowered to detect differences in postoperative outcomes. Recruitment and limited time before surgery were major implementation barriers. No intervention-related adverse events were reported.

Furyk et al. [13] assessed the feasibility of exercise-based prehabilitation in frail patients scheduled for CRC surgery. Of 106 screened patients, only five were randomized, highlighting major recruitment and logistical barriers. Because of the extremely small sample and limited follow-up, no conclusions on effectiveness could be drawn. However, the intervention and functional assessments were safe and well tolerated.

Peng et al. [21] conducted a prospective RCT in 213 patients undergoing elective colorectal surgery, comparing prehabilitation-enhanced ERAS with standard ERAS. The approximately 2-week home-based program included limb strengthening, breathing, and abdominal exercises under therapist supervision. The intervention improved gastrointestinal recovery according to I-FEED, selected QoR-40 domains, and handgrip strength, but did not significantly affect complication rates or length of stay. No serious intervention-related adverse events were reported.

McIsaac et al. [22] performed a double-blind RCT in 204 frail older adults undergoing elective oncologic surgery, including CRC procedures. The remotely supported home-based program included strength, aerobic, flexibility, and nutritional components over a mean period of about 5 weeks. No significant differences were found in the primary outcome (postoperative 6MWT) or in secondary outcomes such as quality of life, disability, complications, or length of stay. However, per-protocol analysis suggested better functional recovery and fewer complications among highly adherent participants. The program was safe and feasible.

Bojesen et al. [23] evaluated multimodal prehabilitation in a prospective RCT involving 36 patients undergoing elective curative CRC surgery. The outpatient program combined supervised HIIT and resistance training, nutritional support, and medical optimization for at least 4 weeks before surgery. Prehabilitation significantly improved early postoperative recovery measured by QoR-15, although no significant differences were found in complications, length of stay, or functional capacity. No intervention-related serious adverse events were observed.

Triguero-Cánovas et al. [24] conducted a prospective RCT in 60 patients with CRC, showing that home-based prehabilitation significantly improved preoperative and early postoperative functional capacity, as reflected by 6MWT and cardiopulmonary fitness measures. Trends toward lower complication rates and shorter hospital stays were observed but were not statistically significant. The intervention was feasible, safe, and well tolerated.

Molenaar et al. [25] conducted an international multicentre RCT in 251 patients undergoing elective resection for nonmetastatic CRC. The 4-week supervised multimodal program included aerobic and resistance training, nutritional optimization, psychological support, and smoking cessation within ERAS care. Prehabilitation significantly reduced severe postoperative complications (CCI > 20) and medical complications, while improvement in postoperative 6MWT did not reach statistical significance. Minor adverse events occurred in a small proportion of participants, but no serious intervention-related events were reported.

Atoui et al. [26] evaluated home-based multimodal prehabilitation in a pilot RCT of 102 patients scheduled for elective CRC resection. The intervention combined exercise, nutritional counseling, and psychological support. Most sleep-related outcomes did not differ significantly between groups, although slight improvement in perceived sleep quality was noted preoperatively, particularly in patients with higher baseline anxiety. The program was feasible and safe.

Cate et al. [14] conducted a prospective single-arm study in 101 patients awaiting CRC surgery. A 3–4-week multimodal program improved aerobic capacity, 6MWT distance, and muscle strength, with the greatest gains observed in patients with the lowest baseline functional capacity. Adherence was excellent and no serious adverse events were reported, although the single-arm design limits conclusions regarding comparative effectiveness.

Gamage et al. [27] presented the protocol of an RCT investigating mindfulness-based trimodal prehabilitation in CRC patients undergoing elective resection. The planned intervention combines home-based exercise, nutritional optimization, psychological coping strategies, and mindfulness training over 4 weeks. As this is a protocol study, no clinical outcome data are yet available.

Gonella et al. [15] evaluated multimodal prehabilitation in frail older CRC patients using a prospective trial with historical controls. The intervention was associated with lower postoperative complication rates, lower complication severity, shorter length of stay, improved 6MWT performance, and reduced anxiety. No intervention-related adverse events were reported, and compliance was high.

Groen et al. [16] assessed community-based multimodal prehabilitation in high-risk CRC patients using a prospective trial with historical controls. Compared with controls, prehabilitation was associated with significantly fewer postoperative complications, fewer multiple complications, lower readmission rates, and better postoperative functional outcomes, including 6MWT, VO_2_max, and muscle strength. The program was safe and showed high adherence.

Ip et al. [28] conducted a feasibility study of a web-based multimodal prehabilitation platform (PREP) in patients awaiting elective CRC surgery. The intervention promoted physical activity, healthy diet, smoking cessation, and psychological coping, with optional dietitian and coaching support. The program was well accepted and feasible, although no clear postoperative quality-of-life benefit was demonstrated. No intervention-related adverse events were reported.

Pesce et al. [29] reported interim results of an RCT evaluating trimodal prehabilitation in 71 patients undergoing elective CRC resection. The 4-week program significantly improved preoperative and postoperative functional capacity, as reflected by 6MWT performance, but did not significantly reduce complication rates or hospital stay in the interim analysis. The intervention was feasible and well tolerated.

van der Hulst et al. [30] conducted an observational cohort study with historical comparison in patients aged ≥75 years undergoing elective CRC surgery. Multimodal prehabilitation did not significantly improve long-term survival, but was associated with fewer hospital admissions during follow-up. No safety concerns were reported.

Yang et al. [31] performed a prospective RCT evaluating exercise-based prehabilitation integrated with ERAS in 95 patients undergoing laparoscopic CRC surgery. The intervention improved postoperative frailty status, short-term recovery quality, earlier ambulation, and bowel recovery, but did not significantly affect complications, length of stay, or 6MWT. No exercise-related adverse events were reported.

Chou et al. [32] conducted a stratified RCT of a resilience-based cancer prehabilitation program in 128 patients with stage 0–III CRC. The intervention combined home-based aerobic exercise, resilience training, and mindfulness-based coping strategies, and continued postoperatively. Per-protocol analysis showed improved resilience, reduced fear of recurrence, and better spiritual well-being, while fatigue, depression, and symptom severity did not differ significantly. The program was feasible and safe.

Danielsson et al. [33] evaluated a home-based high-intensity exercise prehabilitation program in older patients with CRC and low physical fitness. The intervention improved postoperative inspiratory muscle strength, but not 6MWT or chair-stand performance. Minor adverse events occurred, and one transient ischemic attack led to discontinuation; overall, the program was considered feasible and acceptably safe.

van Erven et al. [34] conducted a multicentre RCT assessing multimodal prehabilitation combining structured exercise and targeted nutritional optimization in 67 CRC patients. The intervention significantly improved fat-free mass, appendicular skeletal muscle mass, lower-limb strength, and daily protein intake. No serious intervention-related adverse events were reported, although minor intolerance to protein supplementation occurred in some participants.

Suárez-Alcázar et al. [18] evaluated a multimodal prehabilitation program in a longitudinal cohort study with retrospective controls. The intervention improved chair-stand performance, flexibility, and 6MWT distance, and was associated with a shorter stay in the postoperative recovery unit. Trends toward fewer complications and shorter hospitalization were also observed, although these did not reach statistical significance. The program was feasible, safe, and well tolerated.

**Table 1:** Summary of evidence on the use of prehabilitation in surgically treated CRC patients based on current clinical and scientific studies.

Authors	Study Design	Population	Prehabilitation	Key Findings
Bousquet-Dion et al. [19]	RCT	n = 63, CRC, elderly	Multimodal (exercise + nutrition + psych)	↑ activity, ↔ 6MWT
Karlsson et al. [11]	Feasibility RCT	n = 23, ≥70 y	Home-based exercise	↑ IMT, high adherence
Carli et al. [20]	RCT	n = 110, frail ≥65 y	Multimodal	↔ CCI, ↑ preop function
Northgraves et al. [12]	Feasibility RCT	n = 22	Supervised exercise	↑ TUG, 6MWT
Furyk et al. [13]	Feasibility RCT	n = 5, frail	Exercise	↓ feasibility
Peng et al. [21]	RCT	n = 213	Exercise + ERAS	↑ GI recovery, QoR
McIsaac et al. [22]	RCT	n = 204, frail	Home-based multimodal	↔ 6MWT, adherence effect
Bojesen et al. [23]	RCT	n = 36	Multimodal (HIIT)	↑ QoR-15
Triguero-Cánovas et al. [24]	RCT	n = 60	Home-based exercise	↑ 6MWT, fitness
Molenaar et al. [25]	Multicenter RCT	n = 251	Multimodal	↓ complications
Atoui et al. [26]	Pilot RCT	n = 102	Multimodal	↑ sleep (trend)
ten Cate et al. [14]	PCT	n = 101	Multimodal	↑ fitness, strength
Gamage et al. [27]	RCT protocol	n = 72 (planned)	Trimodal + mindfulness	↑ feasibility
Gonella et al. [15]	PCT + historical	n = 166, frail	Multimodal	↓ complications, LOS
Groen et al. [16]	PCT + historical	n = 100, high-risk	Multimodal	↓ complications, ↑ fitness
Ip et al. [28]	Feasibility PCT	n = 33	Web-based multimodal	feasibility
Pesce et al. [29]	RCT (interim)	n = 71	Trimodal	↑ 6MWT
van der Hulst et al. [30]	OCS	n = 223, ≥75 y	Multimodal	↓ admissions
Yang et al. [31]	RCT	n = 95	Exercise + ERAS	↑ recovery, frailty
Chou et al. [32]	RCT	n = 128	Exercise + resilience	↑ resilience
Danielsson et al. [33]	RCT	n = 52, low fitness	Exercise (IMT)	↑ MIP
van Erven et al. [34]	RCT	n = 67	Exercise + nutrition	↑ muscle mass
Suárez-Alcázar et al. [18]	OCS	n = 60	Multimodal	↑ 6MWT

***Abbreviations:*** 6MWT—6-min walk test; CCI—Charlson Comorbidity Index; CRC—colorectal cancer; ERAS—Enhanced Recovery After Surgery; HIIT—high-intensity interval training; IMT—inspiratory muscle training; LOS—length of stay; PCT—prospective cohort trial; QoR—quality of recovery; QoR-15—Quality of Recovery-15 questionnaire; RCT—randomized controlled trial; TUG—Timed Up and Go test. ↑—improvement/increased parameter; ↓—reduction/decreased parameter; ↔—no significant difference/unchanged parameter.

### 3.2. Effectiveness of Multimodal Interventions

Analysis of the clinical studies included in this state-of-the-art narrative review indicates that multimodal interventions represent the predominant and most extensively investigated prehabilitation model in CRC patients. In the majority of RCTs, programs combining physical training, nutritional support, and (in some studies) educational or psychological components resulted in significant improvements in functional capacity during the preoperative period. Functional outcomes were most commonly assessed using the 6MWT, cardiorespiratory fitness indices, and measures of muscle strength. Notably, these improvements were observed even in short-term programs implemented within limited preoperative timeframes, highlighting the feasibility and clinical potential of prehabilitation under real-world conditions.

The impact of multimodal prehabilitation on hard perioperative outcomes was considerably more nuanced and heterogeneous. While selected multi-center trials demonstrated reductions in severe complication rates or downstream readmissions, these trends were not universally replicated across all major RCTs. This variance demonstrates that prehabilitation cannot be assumed to universally improve short-term surgical outcomes; rather, its clinical efficacy is moderate for functional enhancement and highly variable for perioperative metrics, heavily depending on rigorous patient selection and high compliance.

### 3.3. Patient Safety and Tolerability

Safety and tolerability of prehabilitation were evaluated in the vast majority of included clinical studies and represent one of the most consistent domains of available evidence. Across RCTs, the predominant study design, as well as observational and prospective studies, prehabilitation interventions were generally well tolerated. The incidence of adverse events directly related to physical training or other program components was low. Reported adverse effects were typically mild and transient, rarely leading to discontinuation of the intervention or delays in surgical treatment.

An important aspect of safety was the high level of patient acceptance and adherence to prehabilitation programs. This was particularly evident in home-based or hybrid models, which reduced the need for frequent in-person visits. Studies consistently indicated that appropriate patient education, individualized exercise intensity, and realistic program goals facilitated sustained engagement, even among patients with multiple comorbidities or frailty. Overall, available data confirm that prehabilitation (including multimodal interventions) is a safe component of preoperative preparation for CRC patients and does not increase perioperative risk.

## 4. Discussion

### 4.1. Identification of Prehabilitation Implementation Models

Analysis of the clinical studies indicates that prehabilitation in CRC has been implemented using diverse organizational models, including inpatient, outpatient, home-based, and hybrid programs. The most commonly applied approaches involved multimodal interventions integrated into existing ERAS pathways and delivered over periods ranging from 2 to 6 weeks prior to elective surgery. Increasing attention has been given to home-based programs supported by remote monitoring and patient education, which have demonstrated good feasibility and acceptance, particularly among older patients or those with limited mobility. Clinical evidence suggests that the effectiveness of prehabilitation is not strictly dependent on the setting in which the program is delivered but rather on its structure, degree of individualization, and level of integration within perioperative care pathways.

### 4.2. Determinants of Prehabilitation Effectiveness and Endpoint Heterogeneity

The clinical impact of prehabilitation in patients undergoing CRC surgery is influenced by a multi-faceted matrix of patient-related, intervention-related, and organizational factors. However, a critical divergence must be established between subjective or functional improvements and hard clinical endpoints.

This lack of consistency is directly attributable to substantial discrepancies in intervention dose, duration, and protocol structure, which currently hinders the establishment of a standardized dose–response relationship. Operationalizing prehabilitation requires a precise delineation of its fundamental building blocks: physical exercise (stratified by the FITT principle), nutritional optimization, and psychological support. In the current literature, a clear dose–response effect is obscured by highly variable compliance rates and brief implementation windows. For instance, supervised high-intensity interval training (HIIT) protocols [23] significantly enhance subjective short-term quality of recovery (QoR-15) but do not automatically yield baseline cardiorespiratory fitness gains or reduce LOS when nested within highly optimized, standard ERAS pathways that already minimize hospitalization.

Conversely, home-based, low-intensity interventions [22] deliver a lower physiological dose and often suffer from variable compliance, diluting their statistical impact on hard clinical outcomes unless strict per-protocol adherence is maintained. Defining the threshold where cumulative intervention volume triggers a reduction in complications remains a critical priority that requires trials to uniformly report total completed training hours and exact nutritional intake.

### 4.3. Biological and Physiological Mechanisms Underlying Prehabilitation

To understand the clinical variations observed across trials, the specific biological and physiological pathways modulated by prehabilitation must be explored [35]. The surgical resection of CRC triggers a profound, systemic neuroendocrine stress response characterized by hypermetabolism, muscle catabolism, and a massive surge of pro-inflammatory cytokines, such as interleukin-6 (IL-6) and tumor necrosis factor-alpha (TNF-alpha) [36]. Multimodal prehabilitation counteracts this surgical trauma through three interconnected mechanistic pillars [37].

Structured aerobic and resistance exercises stimulate mitochondrial biogenesis, increase stroke volume, and optimize peripheral tissue oxygen extraction [35]. Elevating maximal oxygen uptake (VO_2max_) and the ventilatory threshold prior to incision broadens the patient’s physiological safety margin, allowing organ systems to withstand prolonged periods of perioperative hypoxia and hemodynamic stress without shifting into cell-damaging anaerobic metabolism [35,38]. Cardiopulmonary exercise testing (CPET) plays an increasing role in this domain, not only as a tool for precise risk stratification and defining baseline functional capacity, but also for prescribing personalized, tailored exercise parameters that directly prevent immediate post-surgical functional decline [39]. Indeed, systematic network meta-analyses confirm that high-intensity interval training (HIIT) acts as a highly effective intervention for boosting VO_2max_ and reducing postoperative complication rates across major abdominal and gastrointestinal oncology cohorts [40].

Regular physical activity induces a systemic anti-inflammatory phenotype, partially mediated by the release of muscle-derived myokines (such as IL-10) which blunt the post-surgical cytokine storm [36]. This attenuation limits endothelial dysfunction and downregulates the profound immune suppression typically seen in the immediate postoperative window, lowering susceptibility to secondary infectious and respiratory complications [35]. Integrating exercise alongside tailored metabolic conditioning helps downregulate the systemic stress pathways activated by advanced localized tissue carcinomas [41].

Malnutrition, cancer-induced cachexia, and underlying sarcopenia severely impair wound healing and respiratory muscle function [7]. Targeted preoperative nutritional intervention, predominantly via whey protein supplementation matched with resistance training, reverses insulin resistance and shifts the patient into a positive nitrogen balance [36]. High-quality protein regimens and targeted dietary strategies directly improve clinical outcomes by attenuating inflammation, boosting cell-mediated recovery, and restoring fat-free skeletal muscle mass [41]. This structural and metabolic reinforcement provides the necessary raw substrates required for swift tissue repair and accelerated early post-surgical mobilization, shielding the patient from the steep functional drops commonly noted at hospital discharge [42].

### 4.4. Implementation Challenges in Healthcare Systems

The implementation of prehabilitation programs in routine clinical practice remains constrained by several systemic and organizational barriers across healthcare systems. Despite increasing clinical evidence supporting its safety and potential effectiveness, prehabilitation is not yet consistently integrated into standard perioperative care pathways.

A critical appraisal of real-world applicability reveals a profound gap between controlled trial protocols and pragmatic clinical settings. In randomized trials, patients are frequently backed by dedicated research coordinators, structured phone alerts, and fully subsidized nutritional supplies, which artificially inflate compliance. In routine public healthcare settings, key challenges include the absence of dedicated funding models, limited availability of multidisciplinary teams (surgeons, dietitians, physiotherapists, and psychologists), and insufficient coordination between surgical, rehabilitation, and primary care services.

Furthermore, the real-world utility of prehabilitation is severely bottlenecked by rigid oncological timelines. In fast-track colorectal cancer pathways designed to minimize surgical delays, the window between diagnosis and resection is frequently restricted to under 14 days, which is physiologically insufficient to achieve the full cardiorespiratory and muscular benefits seen in longer 4-to-6-week trial protocols. Digital and home-based prehabilitation models have emerged as potential solutions to improve accessibility and scalability; however, their real-world effectiveness appears to depend heavily on patient digital literacy, underlying socioeconomic baseline factors, and the presence of continuous clinical supervision. Without addressing these logistical friction points, the widespread efficacy of prehabilitation will remain confined to academic literature rather than real-world surgical workflows.

### 4.5. Research Directions

To advance the clinical utility of prehabilitation, protocols must evolve from purely physiological metrics toward a holistic, patient-centered framework. This requires integrating targeted interventions for stoma preparation and sexual health, which are two areas that severely dictate long-term quality of life but remain isolated from current trial designs. Preoperative stoma optimization should encompass specialized psychological counseling for body image adaptation alongside precise anatomical site marking, which directly correlates with reduced long-term stoma-related complications. Similarly, proactive educational and psychological counseling regarding colorectal-surgery-induced intimate and pelvic floor dysfunction must be normalized during the preoperative window. Framing these deeply personal stressors within an established trimodal prehabilitation team minimizes patient alienation, lowers baseline anxiety, and aligns care directly with the authentic, long-term recovery needs of the patient.

### 4.6. Strengths and Limitations of the Review

This review possesses several distinct strengths, including a comprehensive and up-to-date analysis of primary trials spanning a 10-year period (2015–2025) across five major medical databases. By structuring our analysis around the SANRA framework, we have maintained transparency while highlighting previously overlooked clinical domains like stoma preparation and sexual health.

However, certain limitations must be acknowledged. First, because this study was designed as a narrative state-of-the-art review rather than a formal systematic meta-analysis, a quantitative pooling of data and formal statistical grading of risk-of-bias was not performed. Second, the included literature exhibits significant heterogeneity in exercise duration, nutritional formulas, and definitions of surgical complications, which restricts our ability to define a singular, optimal dose–response model for routine implementation. Final conclusions are also constrained by the pilot or feasibility nature of several smaller trials included in the current literature base.

## 5. Conclusions

Over the past decade, interest in prehabilitation for CRC has increased substantially, reflected by a growing number of clinical studies, predominantly randomized trials, despite considerable heterogeneity in study design, patient populations, and intervention characteristics. Across the included studies, prehabilitation programs were consistently safe and feasible, with low rates of intervention-related adverse events and high patient adherence in both supervised and home-based models. These findings support the clinical applicability of prehabilitation as a component of perioperative care.

Overall, current evidence indicates that multimodal prehabilitation reliably improves preoperative functional capacity, but its influence on short-term surgical outcomes remains mixed. The high variability across trials underscores that future research must move away from generalized applications and focus explicitly on establishing standardized protocols tailored to high-risk subgroups who stand to derive the most definitive clinical benefit.

## Data Availability

No new data were created or analyzed in this study. Data sharing is not applicable to this article.

## Supplementary Materials

The following supporting information can be downloaded at: https://www.mdpi.com/article/10.3390/jcm15114128/s1, Table S1: Detailed characteristics of included studies on prehabilitation in CRC.

## Abbreviations

The following abbreviations are used in this manuscript:

1RM	one-repetition maximum
6MWT	6 min walk test
ADL	activities of daily living
ASA	American Society of Anesthesiologists (physical status classification)
BMI	body mass index
CCI	Comprehensive Complication Index
CHAMPS	Community Healthy Activities Model Program for Seniors
CPET	cardiopulmonary exercise testing
CRC	colorectal cancer
ERAS	Enhanced Recovery After Surgery
HADS	Hospital Anxiety and Depression Scale
Hb	hemoglobin
HIIT	high-intensity interval training
HRQoL	health-related quality of life
I-FEED	Intake, Feeling nauseated, Emesis, physical Exam, Duration of symptoms (gastrointestinal recovery score)
IL-6	interleukin-6
IL-10	interleukin-10
IMT	inspiratory muscle training
IQR	interquartile range
ITT	intention-to-treat
LOS	length of hospital stay
MAAS	Mindful Attention Awareness Scale
METs	metabolic equivalents
mTOR	mammalian target of rapamycin
OCS	observational cohort study
OIT/REA	intensive care unit/post-anesthesia recovery unit
PCT	prospective cohort trial
PP	per-protocol analysis
PR-ERAS	Prehabilitation-Enhanced Recovery After Surgery pathway
PS	performance status
PSS	Perceived Stress Scale
QoR	Quality of Recovery
QoR-9	9-item Quality of Recovery score
RCT	randomized controlled trial
S-ERAS	standard Enhanced Recovery After Surgery
SGA	Subjective Global Assessment
SRT	steep ramp test
TNF-alpha	tumor necrosis factor-alpha
VO_2_max	maximal oxygen uptake
WHO	World Health Organization
WHOQOL-BREF	World Health Organization Quality of Life—BREF version
