# Supplementary material for: Clinical Effectiveness and Safety of Multimodal Prehabilitation in Colorectal Cancer Surgery—A Narrative State-of-the-Art Review of Research and Clinical Trials
Source: J Clin Med. 2026 May 27;15(11):4128. doi: 10.3390/jcm15114128 (PMC13258502; doi:10.3390/jcm15114128)
Supplement: Supplementary file 1 [file jcm-15-04128-s001.zip › jcm-4289765-supplementary.pdf]

Journal of Clinical Medicine
MDPI

Table S1. Detailed characteristics of included studies on prehabilitation in CRC.

Authors, year, journal	Study design	Population (n, age)	Prehabilitation model	Prehabilitation period	Main outcomes	Prehabilitation safety
1. Bousquet-Dion et al., 2018; Acta Oncol. [19]	Prospective RCT	63 patients undergoing elective CRC resection; PREHAB+ n = 37, REHAB n = 26; median age 74 vs 71 years	Multimodal prehabilitation including supervised aerobic and resistance training, home-based exercise, nutritional counseling with whey protein supplementation, and anxiety-reduction strategies vs postoperative rehabilitation	Approximately 3-4 weeks preoperatively within an ERAS pathway at a tertiary university hospital	No significant difference in the primary endpoint (functional walking capacity measured by 6MWT). The prehabilitation group demonstrated higher levels of moderate-to-vigorous physical activity (CHAMPS) and better preservation of functional capacity during the perioperative period	No significant intervention-related adverse events; the program was safe and well tolerated
2. Karlsson et al., 2019; PLoS One [11]	Feasibility RCT	23 older patients scheduled for CRC surgery (≥70 years); 21 participants included in final analysis	Supervised home-based exercise program including respiratory training, functional strength exercises, and aerobic activity performed between supervised physiotherapist visits	Short preoperative period before surgery (median ≈17 days); 2-3 supervised sessions per week plus self-administered exercises	High feasibility and acceptability of the program with very high compliance (~97% supervised sessions); significant improvement in inspiratory muscle strength in the intervention group; limited statistical power for clinical outcomes due to small sample size	No serious intervention-related adverse events were reported; the program was considered safe and feasible in older adults
3. Carli et al., 2020; JAMA Surg. [20]	Single-blind parallel-arm RCT	110 frail patients ≥65 years undergoing CRC resection (Prehab n = 55; Rehab n = 55); mean age 78 ± 7 years	Multimodal prehabilitation including supervised aerobic and resistance training, home-based exercise, individualized nutritional counseling with protein supplementation, and psychological support vs identical multimodal rehabilitation initiated after surgery	Approximately 4 weeks before surgery within a standardized ERAS pathway at two tertiary university hospitals	No significant difference in the primary outcome (30-day Comprehensive Complication Index). Secondary outcomes (overall and severe complications, length of stay, readmissions, functional recovery measured by 6MWT, and PROMs) were also similar between groups; greater preoperative improvement in walking capacity was observed in some prehabilitation participants	No intervention-related adverse events were reported

4. Northgraves et al., 2020; Support Care Cancer [12]	Feasibility RCT	22 patients scheduled for elective CRC surgery (Prehab. n = 11; Standard care n = 11); mean age ~64 years	Individualized functional exercise-based prehabilitation including aerobic cycling training and multi-joint resistance/functional exercises supervised by a strength and conditioning specialist	Mean preoperative intervention period ≈22 ± 7.5 days (range 13–35 days) with three supervised sessions per week	Feasibility outcomes showed recruitment and time-to-surgery as major barriers. Participants receiving prehabilitation demonstrated improvements in several physical function tests (TUG, stair climb test, and 6MWT), whereas standard care showed minimal change; no conclusive effect on postoperative length of stay due to small sample size	No intervention-related adverse events were reported; adherence to the intervention was high (~90% of planned sessions attended)
5. Furyk et al., 2021; Front Rehabil Sci. [13]	Single-blind RCT	5 frail patients ≥50 years scheduled for CRC surgery (screened n = 106; randomized n = 5); only 1 patient completed full follow-up	Supervised exercise-based prehabilitation (strength, balance, and high-intensity interval walking) performed 3 times/week combined with dietary advice vs usual care	4-week preoperative program delivered in a regional tertiary hospital setting	Very low feasibility of conducting an RCT in this population (≈5% recruitment rate). Major barriers included short time to surgery, logistical issues, and physical/psychological burden of frailty. Due to very low completion, no meaningful conclusions regarding clinical efficacy could be drawn	No adverse events related to exercise or functional testing were reported among participating patients; assessments and intervention were well tolerated
6. Peng et al., 2021; Chin Med J (Engl). [21]	Prospective RCT	213 patients undergoing elective CRC surgery; PR-ERAS n = 109, S-ERAS n = 104; age range 16–85 years	ERAS protocol supplemented with structured preoperative rehabilitation exercises including limb strengthening exercises, thoracic and abdominal breathing exercises, and abdominal muscle training performed twice daily under therapist guidance	Mean duration 14.3 ± 5.2 days before surgery; predominantly home-based program with supervision by an anesthesiologist/physiotherapist and pre- and postoperative assessment	Higher proportion of normal gastrointestinal recovery according to I-FEED classification in PR-ERAS group (79.0% vs 64.3%); improved postoperative QoR-40 domains (life ability and physical well-being at 72 h) and greater handgrip strength; no significant difference in length of hospital stay or overall complication rates	No exercise-related injuries or serious adverse events were reported; incidence of bowel-related and other complications within 30 days was comparable between groups

https://doi.org/10.3390/jcm15114128

7. McIsaac et al., 2022; Br J Anaesth. [22]	Double-blind RCT	204 frail patients ≥60 years undergoing elective oncologic surgery (102 prehabilitation vs 102 control); mean age 74 years	Remotely supported home-based exercise prehabilitation including strength training (10 exercises), aerobic training (~20 min moderate-intensity activity), flexibility exercises, and nutritional guidance vs standard care with written physical activity and nutrition recommendations	≥3 weeks between enrolment and surgery; mean participation ~5 weeks	NNo significant difference in the primary endpoint (6MWT distance at first postoperative visit; adjusted mean difference 14 m, 95% CI -26 to 55); no significant differences in QoL, disability, complications, or length of stay; per-protocol analysis (≥80% adherence) suggested improved functional recovery (6MWT +76 m) and fewer complications	No study-attributable adverse events during the prehabilitation period; postoperative falls and complications were comparable between groups
8. Bojesen et al., 2023; BJS Open [23]	Prospective RCT	40 patients with CRC and WHO performance status I–II scheduled for elective curative surgery; 36 patients included in final analysis (prehabilitation n = 16, control n = 20); mean age 79 years	Multimodal prehabilitation including supervised high-intensity interval training (HIIT) on a cycle ergometer combined with resistance training (3×/week), nutritional counseling with protein supplementation and vitamins, and medical optimization prior to surgery vs standard care	Minimum 4 weeks before surgery (median 32 days intervention vs 24 days control); outpatient program integrated with ERAS pathway	Significant improvement in early postoperative recovery measured by QoR-15 during the first 3 postoperative days (overall treatment effect +21.9 points; 95% CI 4.5-39.3); no significant differences in postoperative complications, length of stay, or functional capacity measures between groups	Serious adverse events occurred in both groups during the preoperative period but none were related to the prehabilitation intervention; the program showed high adherence and was considered safe
9. Triguero-Cánovas et al., 2023; Support Care Cancer [24]	Prospective RCT	60 patients with CRC scheduled for elective surgery; randomized 1:1 (prehabilitation n = 30, standard care n = 30)	Home-based prehabilitation program including structured physical exercise aimed at improving cardiopulmonary capacity and functional performance, individualized according to cardiopulmonary exercise testing (CPET), with monitoring of training and functional capacity (6MWT)	Preoperative period between diagnosis and surgery (several weeks); assessments at diagnosis, preoperatively, and 6-8 weeks after surgery	Significant improvement in functional capacity in the prehabilitation group (increase in 6MWT distance +78.9 m preoperatively and +68.9 m at 6-8 weeks postoperatively); improved cardiopulmonary fitness measured by ergospirometry (increase in METs); trends toward lower postoperative complications (17.4% vs 33.3%) and shorter hospital stay, though not statistically significant	No intervention-related adverse events reported; the home-based program was feasible and well tolerated

10. Molenaar et al., 2023; JAMA Surg. [25]	International multicenter RCT	251 adults undergoing elective resection of nonmetastatic CRC (prehabilitation n = 123, standard care n = 128); median age 69 years	Supervised multimodal prehabilitation including high-intensity interval aerobic training (cycle ergometer), resistance training targeting major muscle groups, individualized nutritional optimization with protein supplementation, psychological support, and smoking cessation when indicated; integrated with ERAS perioperative care	4-week supervised preoperative program with exercise sessions 3 times per week	Significant reduction in severe postoperative complications (CCI >20: 17.1% vs 29.7%; OR 0.47, p = 0.02) and fewer medical complications in the prehabilitation group; postoperative functional recovery favored prehabilitation, although improvement in 6MWT at 4 weeks did not reach statistical significance	Minor adverse events occurred in 5.7% of participants (e.g., lightheadedness during exercise or intolerance to protein supplements), but no serious intervention-related adverse events were reported
11. Atoui et al. J Behav Med. [26]	Pilot RCT	102 patients scheduled for elective CRC resection (prehabilitation n = 50, standard care n = 52); mean age 65 years; 48.3% female	Home-based multimodal prehabilitation including supervised physical exercise, nutritional counseling, and psychological support delivered during the preoperative period vs standard care	Preoperative period until surgery with follow-up assessments preoperatively and at 4 and 8 weeks after surgery	No significant differences between groups in most subjective and objective sleep parameters; slight improvement in perceived sleep quality preoperatively in the prehabilitation group; patients with high anxiety showed significant improvement in sleep duration over time	The intervention was feasible with acceptable recruitment (54%) and retention (72%); no intervention-related adverse events were reported
12. ten Cate et al., 2024; Acta Oncol. [14]	Single-arm PCT	101 adults scheduled for CRC surgery; mean age 69.7 ± 12.7 years	Multimodal prehabilitation including supervised endurance and resistance training, individualized dietary consultation with protein and vitamin supplementation, smoking cessation support, and psychological support	Approximately 3-4 weeks before planned surgery; outpatient preoperative program	Significant improvements in aerobic capacity (steep ramp test +28.3 W; +16.7%), functional walking capacity (6MWT +37.5 m; +7.7%), and muscle strength (1RM +16–32%); greatest improvements observed in patients with the lowest baseline functional capacity	No serious adverse events related to the program were reported; adherence to scheduled training sessions was very high (median 100%)
13. Gamage et al., 2024; BMJ Open [27]	RCT (protocol)	Planned sample: 72 adult patients with CRC undergoing elective CRC resection; randomized 1:1 (36 intervention vs 36 control); age ≥18 years	Mindfulness-based tri-modal prehabilitation: standard tri-modal program (daily home-based physical exercise, individualized nutritional intervention, and psychological coping strategies) plus mindfulness training; control group tri-modal prehabilitation without mindfulness	4-week preoperative program delivered predominantly in a home-based format; ERAS applied perioperatively	Primary: functional capacity measured by the 6MWT; secondary: nutritional status (BMI, albumin, HGb, handgrip strength), psychological status (HADS, MAAS, PSS, WHOQOL-BREF), and stress biomarkers (β-endorphin and cortisol)	Safety monitoring panel established; no safety signals reported at the protocol stage; the multimodal program is considered feasible with monitoring of adherence and adverse events

14. Gonella et al., 2024; Eur J Surg Oncol. [15]	PCT with historical control comparison	166 frail patients ≥65 years with CRC (prehabilitation n = 36; historical control n = 130); frailty defined as functional reserve <6 METsage approx. 80 years	Multimodal prehabilitation including physiotherapy-based exercise training, nutritional optimization, and psychological support; program delivered either in-person or via tele-prehabilitation; integrated with ERAS and followed by structured postoperative rehabilitation coordinated by a case-manager nurse	Median 3 weeks preoperatively (range 2–4 weeks)	Lower 30-day postoperative complication rate in the prehabilitation group (31% vs 53%, p = 0.02), reduced complication severity (lower CCI), shorter hospital stay (median 4.5 vs 6 days), improved functional capacity (mean increase in 6MWT ≈86 m), and reduced anxiety scores	No adverse events reported during the prehabilitation period; program compliance 91%
15. Groen et al., 2024; Eur J Surg Oncol. [16]	PCT with historical control	100 high-risk patients with CRC (aged ≥70 years or ASA class III-IV); 50 in the prehabilitation cohort and 50 in the non-prehabilitation cohort	Community-based multimodal prehabilitation including supervised high-intensity interval and resistance training (3×/week), home-based low-intensity exercise (4×/week), individualized nutritional optimization with high-protein diet and whey supplementation, psychological support, smoking/alcohol cessation, and optimization of comorbidities	≥3 weeks before surgery; supervised sessions in a community physiotherapy practice with additional home training	Significantly lower postoperative complication rate in the prehabilitation group (32.7% vs 58.0%, p = 0.015), fewer multiple complications, and lower 90-day readmission rate (0% vs 12%); functional capacity significantly improved postoperatively (6MWT, VO_2max, and muscle strength tests)	No adverse events reported during the prehabilitation program; 98% of participants completed ≥3 weeks of the intervention
16. Ip et al., 2024; Colorectal Dis. [28]	Feasibility PCT	33 patients scheduled for elective CRC surgery recruited; 28 enrolled; 25 completed baseline and 21 completed follow-up; mean age 52.6 ± 14.2 years	Web-based multimodal prehabilitation programme (PREP) co-developed with patient partners, including four components: physical activity promotion, healthy diet guidance, smoking cessation, and psychological coping/stress-management support; optional access to dietitian counselling and health coaching	≥2 weeks before surgery (typically 2–3 weeks between enrolment and operation)	High acceptability and engagement: 71% visited the website during the preoperative period and 76% reported that the programme provided appropriate support; most patients set lifestyle modification goals (healthy eating 86%, physical activity 81%, stress reduction ≈70%); feasibility demonstrated but no clear improvement in postoperative QoL (EQ-5D-5L slightly decreased at 3-month follow-up)	No adverse events related to the programme were reported; intervention considered feasible and well tolerated

https://doi.org/10.3390/jcm15114128

17. Pesce et al., 2024; Surg Endosc. [29]	RCT (interim analysis)	71 patients undergoing elective CRC resection; prehabilitation n = 35 vs control n = 36; mean age approx. 68–70 years	Trimodal prehabilitation: supervised exercise training (interval and resistance training with CPET-based intensity prescription and additional home aerobic exercise), nutritional optimization (dietitian-guided protein supplementation, multivitamins, omega-3 fatty acids), and psychological support (psycho-oncology assessment and coping strategies); both groups managed within an ERAS pathway	4 weeks before surgery; supervised sessions three times per week combined with home-based aerobic training	Significant improvement in functional capacity in the prehabilitation group: preoperative increase in 6MWT distance (+96 m vs control); benefits maintained at 4 and 8 weeks postoperatively (≈+103 m and +90 m respectively); no significant differences in postoperative complications or hospital length of stay in the interim analysis	The program was feasible and well tolerated; no increase in postoperative complications or perioperative risk compared with standard care
18. Van der Hulst et al., 2024; Eur J Surg Oncol. [30]	OCS (historical comparison)	223 patients aged ≥75 years undergoing elective CRC surgery; standard care n = 137 vs prehab. n = 86	Multimodal prehabilitation implemented as part of perioperative care, including physical training, nutritional optimization, and general functional preparation before surgery	Preoperative program implemented prior to surgery (exact duration not specified); comparison of cohorts treated before vs after implementation of the program	No significant difference in long-term survival at 6 years (59.3% vs 51.1%; p = 0.167); significantly fewer hospital admissions during follow-up in the prehabilitation group (OR 0.43; 95% CI 0.24-0.77)	No safety concerns reported; prehabilitation implementation was not associated with increased perioperative risk
19. Yang et al., 2024; Front Oncol. [31]	Prospective RCT	95 patients undergoing elective laparoscopic CRC surgery; PR-ERAS n = 50 vs S-ERAS n = 45; mean age approx. 60–64 years	Exercise-based prehabilitation integrated with ERAS (PR-ERAS): twice-daily supervised low-intensity exercises including upper limb, breathing, and lower limb exercises performed preoperatively and continued perioperatively, compared with standard ERAS care	Mean preoperative period ≈13 days between enrollment and surgery	Improved postoperative frailty status (lower Fried Frailty Phenotype scores on postoperative day 7), improved short-term recovery quality (higher QoR-9 scores), earlier ambulation and earlier return of bowel function; no significant differences in postoperative complications, length of stay, or 6MWD	No exercise-related adverse events reported; no injuries, falls, or surgery delays attributable to the program
20. Chou et al., 2025; Eur J Oncol Nurs. [32]	Stratified, parallel-group RCT	128 patients with newly diagnosed stage 0-III CRC scheduled for surgery (intervention n = 63, control n = 65); mean age 59.1 ± 11.6 vs 62.8 ± 11.3 years	Resilience model–based cancer prehabilitation integrating physical exercise (home-based moderate-intensity aerobic activity ≈150 min/week monitored with wearable device), psychological resilience training, and mindfulness-based coping strategies delivered through in-person and remote sessions	Program initiated approximately 2 weeks before surgery and continued postoperatively for a total of 12 weeks with follow-up assessments up to 12 months	Per-protocol analysis showed significantly greater improvement in overall resilience at 12 months (β = 11.723, p = 0.003); significant reductions in fear of cancer recurrence and improved spiritual well-being; no significant differences in symptom severity, fatigue, or depression	No intervention-related adverse events reported; program adherence monitored via wearable devices and weekly follow-up

21. Danielsson et al., 2025; J Prim Care Community Health [33]	Multicentre RCT (parallel-group)	52 patients ≥65 years with CRC and low physical fitness scheduled for elective surgery; intervention n = 27 vs control n = 25; mean age 77 ± 6 years	Home-based high-intensity exercise prehabilitation supervised by physiotherapists including inspiratory muscle training (IMT), interval aerobic training (e.g., walking or stair climbing), and functional strength exercises targeting lower extremity strength (chair rise, step-up)	2–3 weeks before surgery; median duration 14 days with at least six supervised sessions plus additional unsupervised home training	Significant improvement in postoperative inspiratory muscle strength (MIP) in the intervention group; no significant differences between groups in functional capacity (6MWT) or lower-extremity strength (30-s chair stand test); intervention group showed higher levels of high-intensity physical activity preoperatively	Few minor adverse events reported (e.g., transient dizziness, musculoskeletal discomfort); one transient ischemic attack led to discontinuation of the intervention; overall program considered feasible and safe
22. van Erven et al., 2025; Clin Nutr ESPEN [34]	Multicentre RCT	67 adults with CRC scheduled for elective surgery (standard care n = 34; multimodal prehabilitation n = 33); mean age 71 ± 10 years	Multimodal prehabilitation including supervised aerobic and resistance training (3 sessions/week), individualized dietary counseling targeting ≥1.5 g/kg/day protein intake, whey protein supplementation (30 g twice daily around exercise and before sleep), vitamin D and multivitamin supplementation	Approximately 4 weeks before surgery	Prehabilitation significantly increased fat-free mass (+0.9 kg vs +0.2 kg; $p = 0.017$), appendicular skeletal muscle mass (+0.5 kg; $p = 0.007$), and muscle strength (1RM leg press +29 kg vs +10 kg; $p < 0.001$); protein intake increased significantly (+43 g/day; $p < 0.001$); 47% achieved target protein intake ≥1.5 g/kg/day	No serious adverse events related to the nutritional intervention reported; minor intolerance to protein supplements (e.g., gastrointestinal discomfort, taste issues) observed in a small proportion of participants
23. Suárez-Alcázar et al., 2025; Healthcare (Basel) [18]	Longitudinal OCS with retrospective control	30 patients with CRC undergoing elective surgery (intervention group n = 30; matched control group n = 30); mean age 64.8 ± 9.1 years	Multimodal prehabilitation including four components: health education and self-care (nurse-led), nutritional counseling, psychological support, and supervised physical exercise (aerobic and resistance training twice weekly)	Approximately 3-4 weeks before surgery	Significant improvements in physical and cardiorespiratory fitness: increased chair-stand performance, improved flexibility (sit-and-reach), and longer distance in the 6MWT ($p = 0.001$). Trends toward fewer postoperative complications and shorter hospital stay; significant reduction in time spent in the postoperative resuscitation unit ($p = 0.009$)	No serious adverse events related to prehabilitation reported; the intervention was feasible and well tolerated by participants

Abbreviations: 6MWT – 6-minute walk test; 1RM – one-repetition maximum; ADL – activities of daily living; ASA – American Society of Anesthesiologists (physical status classification); BMI – body mass index; CCI – Comprehensive Complication Index; CHAMPS – Community Healthy Activities Model Program for Seniors; CPET – cardiopulmonary exercise testing; CRC – colorectal cancer; ERAS – Enhanced Recovery After Surgery; HADS – Hospital Anxiety and Depression Scale; Hb – hemoglobin; HRQoL – health-related quality of life; I-FEED – Intake, Feeling nauseated, Emesis, physical Exam, Duration of symptoms (gastrointestinal recovery score); IMT – inspiratory muscle training; IQR – interquartile range; ITT – intention-to-treat; LOS – length of hospital stay; MAAS – Mindful Attention Awareness Scale; METs – metabolic equivalents; OCS – observational cohort study; OIT / REA – intensive care unit / post-anesthesia recovery unit; PCT – prospective cohort trial; PP – per-protocol analysis; PR-ERAS – prehabilitation-enhanced Enhanced Recovery After Surgery pathway; PS – performance status; PSS – Perceived Stress Scale; QoR / QoR-9 – Quality of Recovery / 9-item Quality of Recovery score; RCT – randomized controlled trial; SGA – Subjective Global Assessment; SRT – steep ramp test; S-ERAS – standard Enhanced Recovery After Surgery; WHO – World Health Organization; WHOQOL-BREF – World Health Organization Quality of Life – BREF version; VO_2max – maximal oxygen uptake.

https://doi.org/10.3390/jcm15114128
